# A Study on Long-Term Oxidation and Thermal Shock Performance of Nanostructured YSZ/NiCrAlY TBC with a Less Dense Bond Coat

**DOI:** 10.3390/ma16155294

**Published:** 2023-07-27

**Authors:** Teodor-Adrian Badea, Mihaela-Raluca Condruz, Alexandru Paraschiv

**Affiliations:** 1 Romanian Research and Development Institute for Gas Turbines COMOTI, 220D Iuliu Maniu Av., 061126 Bucharest, Romania; teodor.badea@comoti.ro (T.-A.B.); alexandru.paraschiv@comoti.ro (A.P.); 2 Materials Science and Engineering Faculty, University Politehnica of Bucharest, 060042 Bucharest, Romania

**Keywords:** thermal barrier coatings, thermally grown oxide, porosity, thermal shock

## Abstract

This paper focused on studying the performance of a nanostructured thermal barrier coating (TBC) system deposited by APS, which had a bond coat with inter-lamellar porosities that resulted during the manufacturing process. The higher porosity level of the bond coat was studied as a possible way to keep the thickness of the TGO under control, as it is distributed on a higher surface, thereby reducing the chance of top-coat (TC) spallation during long-term oxidation and high-temperature thermal shock. The TBC system consisted of nanostructured yttria partially stabilized zirconia (YSZ) as a top coat and a conventional NiCrAlY bond coat. Inter-lamellar porosities ensured the development of a TGO distributed on a higher surface without affecting the overall coating performance. Based on long-term isothermal oxidation tests performed at 1150 °C, the inter-lamellar pores do not affect the high resistance of nanostructured TBCs in case of long-term iso-thermal oxidation at 1150 °C. The ceramic layer withstands the high-temperature exposure for 800 h of maintaining without showing major exfoliation. Fine cracks were discovered in the ceramic coating after 400 h of isothermal oxidation, and larger cracks were found after 800 h of exposure. An increase in both ceramic and bond-coat compaction was observed after prolonged high-temperature exposure, and this was sustained by the higher adhesion strength. Moreover, in extreme conditions, under high-temperature thermal shock cycles, the TBC withstands for 1242 cycles at 1200 °C and 555 cycles at 1250 °C.

## 1. Introduction

The progress in gas turbine propulsion systems was made possible by the constant increase in the temperature at the turbine entry (commonly abbreviated as TET—turbine entry temperature, or as TIT–turbine inlet temperature). Behind this progress stands the technological advances in the fields of material science and manufacturing technologies. The development of new refractory materials, the development of internal cooling systems for components, and thermal barrier coatings (TBCs) are the main reasons for this incredible increase in TET and the enhanced performance of gas turbines. Nowadays, TBCs are common elements of gas turbine components, and many extensive studies have been published in this field, where information regarding the characteristics of TBCs and manufacturing methods can be found [1,2,3].

The main goal of TBCs is to protect the component against high-temperature loads and environmental loads, such as hot corrosion and erosion. TBCs consist mainly of three layers, starting from the substrate (which is usually a refractory material, such as a nickel-based superalloy) to the top layer: a bond coat (BC), a thermally grown oxide (TGO), and a ceramic top coat (TC). Multiple-layered TBCs were also developed more recently as a solution to increase their durability and performance [4,5,6,7].

Each layer of the TBC has its characteristics and purpose; for example, the BC is an essential component as it enhances the adhesion of the TC to the substrate. The TGO is formed at the interface BC-TC, as the oxygen flow penetrates the porous TC and reacts with the chemical compounds of the BC. The layers in the TBC system have different thicknesses. The BC layer typically ranges from 50–150 µm, and the TGO can vary from a few nanometers up to 5 µm (it has been reported that at more than 6 µm spallation, failure happens [8]). On the other hand, the TC has a thickness in the range of 200–300 µm [9,10].

The properties of the layers are influenced by the manufacturing process that is used. Over the years, several methods have been developed and applied to manufacture TBCs, such as atmospheric plasma spraying (APS), electron beam-physical vapor deposition (EB-PVD), high-velocity oxygen fuel (HVOF) spraying, detonation spraying, ion plating, magnetron sputtering, laser cladding, arc evaporation, ion-beam assisted deposition, chemical vapor deposition (CVD), or combined processes, such as PS-PVD, and newer processes, including suspension plasma spraying (SPS), radio frequency plasma-assisted physical vapor deposition, and flame-assisted vapor deposition [11,12,13].

In terms of the materials used for the TBCs, several materials have been tested over the years and some presented outstanding properties [14,15], and these are the ones that are commonly used. In the case of the BC, two common systems are preferred: the Pt-modified aluminides (NiAl) and the M-Cr-Al-X alloy (where M = Ni and/or Co, X = Y, H, Si and/or Ta) [16,17,18]. It is believed that the addition of Pt to β-NiAl can improve the oxidation performance of the coating due to enhanced adhesion between the bond coat and the substrate [19]. A distinct characteristic of Pt-modified aluminides is that when they start to become damaged during high-temperature thermal cycling, their surface starts to undulate (this phenomenon is called “rumping”). This phenomenon proceeds the delamination of TC [16]. Studies were made in the field of optimizing the chemical composition of Pt-modified coating, and also on coatings modified with other elements, such as Pd, Pt, Rh, Hf, etc. [20,21]. The other conventional BC is the M-Cr-Al-X alloy, mainly Ni-Cr-Al-Y or Ni-Co-Cr-Al-Y. It consists of a solid solution γ-Ni matrix and other phases, such as β-NiAl, γ′-Ni_3_Al, and σ-(Cr, Co) phases. To these compounds, up to 1%wt. Y is added to improve the adhesion of the TC [16].

In the case of the TC, it has been reported that several materials can be suitable for TBCs, including TiO_2_, ZrO_2_, Al_2_O_3_, kaolinite, and mineral oxides, such as pyrochlores (A_2_ + B_2_ + O_7_), as well as garnets, monazites, xenotimes, perovskites (ABO 3), rare earth magnesium hexaaluminate (REMHA), etc., but the most common material used is yttria partially stabilized zirconia (YSZ) [7,22,23,24,25,26,27,28,29,30]. The main characteristics that promote YSZ as a good ceramic coating are low thermal conductivity, high dielectric constant, high fracture toughness, chemical inertness at high temperatures, and great resistance to thermal shock [31]. The stabilizer maintains the tetragonal phase of zirconia at room temperature, and it transforms into a monoclinic phase under external stress, causing a volumetric expansion that acts as a crack propagation holder. The main disadvantage of YSZ is its limitation caused by long-term exposure at temperatures over 1200 °C when spallation appears [7]. Studies were made to improve the YSZ properties by dispersing fine nanoparticles, forming nanostructured YSZ that can be observed as nanozones with unmelted or partially melted particles [16,32,33].

As already mentioned, during exposure to high temperatures, the oxygen diffuses through the pores of the TC, and at the interface, TC-BC, a TGO scale, develops due to the oxidation of the BC [34,35,36]. The TGO has a crucial role in the lifespan of the TBCs, as it promotes the spallation of the TC in certain conditions. The oxidations of M-Cr-Al-X result in the development of different types of Al_2_O_3_, with the stable α-Al_2_O_3_ being preferred [18]. If a continuous scale of Al_2_O_3_ is formed on the BC, the TGO acts as a diffusion barrier, suppressing the growth of other detrimental oxides during further oxidation in service. However, if an inconsistent layer of TGO is formed on the BC consisting of several oxides and spinels, this can lead to TBC failure due to spallation [37,38]. 

The TBC can fail by three main mechanisms or by combinations of them: (1) spallation, (2) damage by impact with different objects or particles, and (3) the development of cracks due to interaction with calcium-magnesium-aluminosilicate (CMAS). The usual failure is caused by spallation resulting after fractures that are formed in the TC during high-temperature sintering, or fractures resulting due to TGO growth that embrittle the BC as the alloying elements form more oxides [13].

Apart from the studies regarding different types of materials used as BC or TC, research was conducted in terms of the enhancement of the durability of the TBC by using functionally-graded materials (FGM), and thus becoming functionally-graded TBC or FG-TBC [39]. Another method to improve the durability of TBCs is to deposit different ceramic layers with a gradual increase in porosity in order to reduce thermal conductivity without decreasing the mechanical integrity or adhesion between the layers [40,41,42,43,44].

The present study aimed to assess the performance of a nanostructured thermal barrier coating system deposited by APS that has a bond coat with inter-lamellar porosities resulting from the manufacturing process. This is an unconventional approach, as the presence of a higher porosity level provides a lower densification of the bond coat, which is usually detrimental for the TBCs. The higher porosity level of the bond coat was studied as a possibility to keep the thickness of the TGO under control, as it is distributed on a higher surface, thereby reducing the chance of TC spallation during long-term oxidation and high-temperature thermal shock.

## 2. Materials and Methods

### 2.1. Specimen Manufacturing

**Figure 1:**
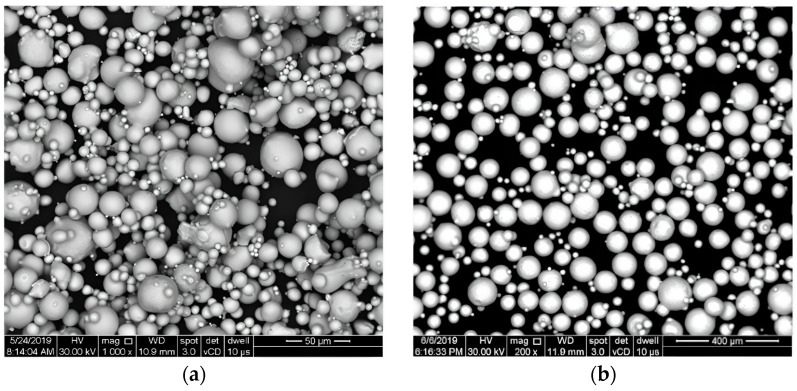
SEM images with the powders used for TBC system: (**a**) the Amperit 413 NiCrAlY powder; (**b**) the Nanox^TM^ Powder S4007.

For this study, 50 × 30 × 4 mm^3^ plates of Nimonic 75 Ni-base superalloy were used as a substrate for the deposition of a TBC system. The bond coat selected was a NiCrAlY powder (Amperit 413) with particle size in the range of 5–45 µm, purchased from H.C. Starck, Golsar, Germany. For the ceramic coating, Nanox^TM^ Powder S4007 was selected, which is a yttria nanostructured zirconia powder (7%wt. Y_2_O_3_, 93%wt. ZrO_2_) from Inframat^®^ Corporation, Farmington, CT, USA, with an agglomerated powder size in the range of 15–150 µm. Scanning electron microscopy images (SEM) with the used powders are presented in Figure 1. The chemical composition of the powders used is presented in Table 1.

The layers were deposited by the APS method, using the Metco F4MB plasma gun (Oerlikon Metco, Wohlen, Switzerland), operated by a 6-axes robot (Kuka KR 150, KUKA ROBOTER GmbH, Augsburg, Germany). The spray parameter set was selected based on the prior experience of the authors with the equipment and the process, and the deposition parameters are presented in Table 2.

It is known that in the case of thermally sprayed coatings, the porosity level is governed by the selection of the process parameters [45], and it was reported that in case of APS, the porosity can vary from below 2 vol.% to greater than 20 vol.%. [46].

### 2.2. Experimental Investigations 

Long-lasting high-temperature oxidation tests were conducted on coated plates. Plates of 15 × 15 × 4 mm^3^ were cut, and they were placed in alumina crucibles previously cleaned with isopropyl alcohol. The tests consisted of cycles of isothermal oxidation heat treatment at 1150 °C. The tests were performed in an electrical Nabertherm LH 30/14 furnace (Nabertherm GmbH, Lilienthal/Bremen, Germany, Tmax = 1400 °C). The oxidation process was realized over 8 cycles of 100 h each until 800 h of total exposure time was reached. An oxidation cycle consisted of heating the crucible with the specimen from room temperature to 1150 °C, using a heating rate of 10 °C/min and a 100-h holding temperature, followed by cooling until room temperature was achieved.

**Figure 2:**
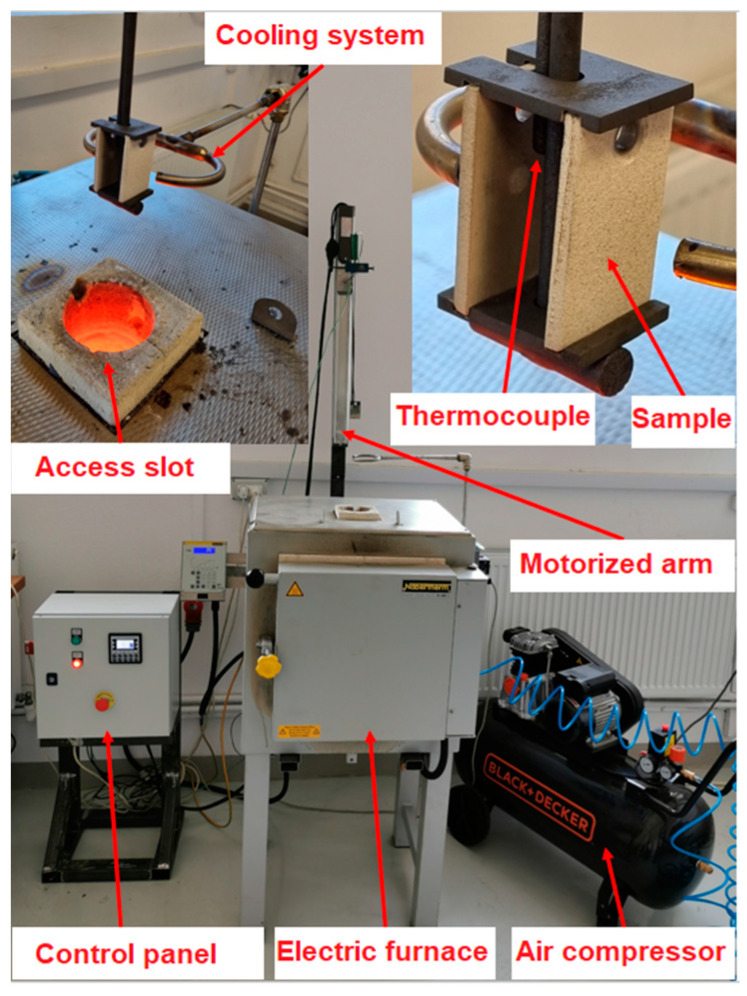
Experimental equipment used for thermal shock.

Cyclic thermal shock tests were also performed on coated specimens by furnace cycle tests (FCT). They were carried out by using testing equipment that was developed in-house, consisting of the same electrical furnace Nabertherm LH 30/14 used for isothermal oxidation testing, which has an access slot on top, a motorized axis controlled by a programmable logic controller (PLC), a sample holder, a thermocouple type S, and a compressed air-cooling system. The experimental equipment is presented in the image from Figure 2. 

Two temperatures were used for the cyclic thermal shock, 1200 °C and 1250 °C. The testing regimen was heating the furnace to 1200 °C or 1250 °C, sliding in the specimens, heating for 60 s, maintaining at a temperature for 100 s, extracting the specimens, and cooling them down with compressed air (3.5 bars) for 120 s until the temperature of 90 °C was registered at the specimen level. The same cycle was also realized at 1250 °C. The experiments were stopped when 30% of the active surface of the ceramic coating was damaged by spallation. The thermal shock tests were performed only to show the durability of the TBC with the porous bond layer in time. 

The microstructural analysis of the specimens was realized by scanning electron microscopy (SEM) using an F50 Inspect SEM equipped with an energy-dispersive X-ray spectrometer (EDS) EDAX APEX 2i, SDD Apollo X detector (FEI Company, Brno, Czech Republic) and EDAX Genesis software V6.29 (EDAX Inc. AmetekMAD, Mahwah, NJ, USA). 

Backscattered electron images with a low-voltage high-contrast detector (vCD detector, FEI Company) were captured to highlight the chemical composition differences at the interface between the oxide layer and the substrate. The element distribution maps and micro-compositional analysis on two micro-areas and element maps were performed by SEM-EDS on the vCD images obtained on the cross-section of the specimens using an acceleration voltage of 30 kV, a take-off angle of 35.6°, a spot size of 3.5 and 4.5 nm, and a working distance of 11, 12.3, 12.6, and 14.9 mm. The analysis was performed on initial state specimens before starting the oxidation cycles, on specimens after 400 h of isothermal exposure, and on specimens after 800 h of isothermal exposure.

**Figure 3:**
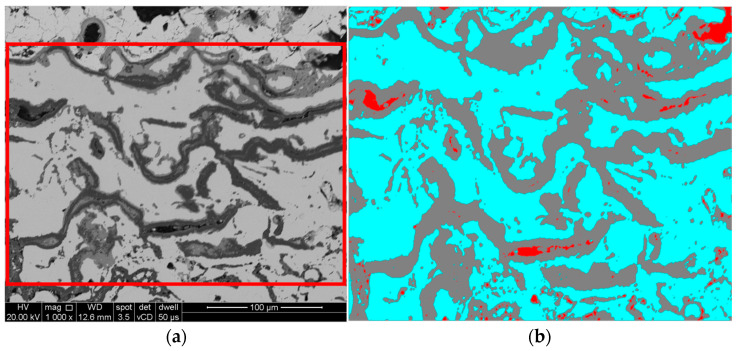
Example of trinarization based on SEM images: (**a**) SEM image with the analyzed surface (marked with the red border); (**b**) processed image based on the SEM image.

The quantitative measurements of the pores and the quantity of TGO were performed using the image analysis software SCANDIUM (Olympus Soft Imaging Solutions GmbH, Munster, Germany) by trinarization technique of 10 SEM images for each case. Figure 3 shows an example with the analyzed area affected by the thermally grown oxide (TGO) and trinarization for quantitative measurement of both pores (red color) and TGO (grey color), based on the image scale and phase distribution. 

The adhesion between the ceramic coat and the bond coat was assessed by scratch testing on the cross-section of specimens in the initial state after 400 h and 800 h of oxidation exposure. For this analysis, the Scratch Tester RST3 (Anton Paar, Graz, Austria) was used, applying the cone area projected method and measuring it with a Carl Zeiss Axio Vert.A1 MAT optical microscope. The strength of the coating was determined using the following parameters––scratch distance: 1.25 mm; the minimum distance between scratches: 1 mm; indenter speed: 3 mm/min; constant pressure force: 40 N; number of tests: 5, with the results reported as an average of these 5 tests; and Rockwell indenter radius: 200 μm.

## 3. Results and Discussion

**Figure 4:**
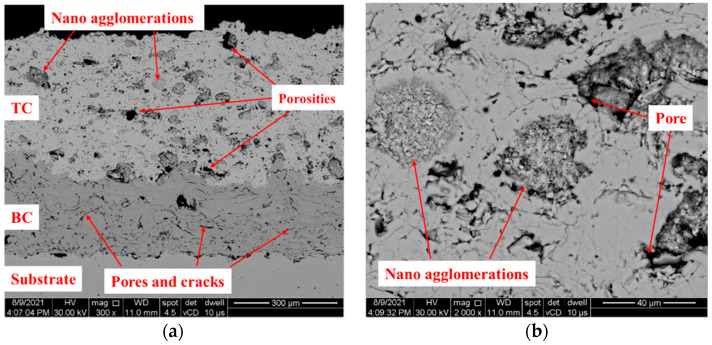
SEM images highlighting the microstructural characteristics of the TBC system (**a**) and the nanopowder agglomerations in the ceramic coating (**b**).

The SEM analysis was performed in the cross-section of the specimens before starting the experimental procedure in order to highlight the initial characteristics of the coating. Figure 4 shows representative SEM images with the TBC coating constitutive layers, and all three distinct areas can be observed: the substrate, the bond coat, and the top ceramic coating. The bond coat presents inter-lamellar flat pores between deposited layers as a result of the voids that are produced between the molten particles’ interaction with the previously partially solidified layer, and this was also reported in the review of Odhiambo [45], where the porosity formation mechanisms are described. The porosity of the bond coat measured in the initial state (Table 3) was 14.42%, which is a value that can be obtained using the APS method (as presented in the range mentioned in [46]). Moreover, the SEM images revealed a good adhesion of the ceramic top coat to the bond coat.

Nano-agglomerations of particles from the yttria nanostructured zirconia powder were observed in the top ceramic layer, as can be seen in the image from Figure 4b.

**Figure 5:**
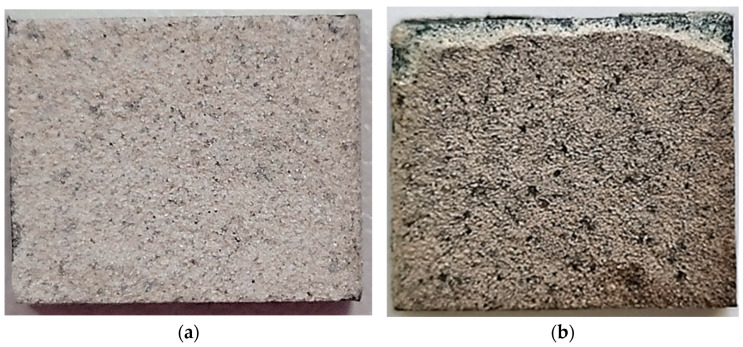
Representative images with the specimens, before (**a**) and after (**b**) 8 cycles at 1150 °C.

Nano-agglomerations were also observed by Xie et al. [32] and by Lima et al. [33], with the last one using the same Nanox powder as we used in the present study. This is a common behavior of nanoparticles, as they tend to agglomerate and to form aggregates due to various reasons, such as high surface energy or electrostatic interactions [47,48]. The isothermal oxidation of specimens revealed the durability of the TBC even after eight cycles of 100 h each of exposure (a total of 800 h). The ceramic layer withstood the high temperature for eight cycles, and showed slight exfoliation at the specimens’ edges (Figure 5b), but it showed an overall good appearance. 

Huang et al. [49] studied the influence of lamellar interface roughness on coating resistance, and they concluded that coatings fabricated with coarse YSZ powders have rough inter-lamellar surfaces, yet these powders have better thermal shock and erosion resistance than those manufactured using finer powders. In the present study, a YSZ powder (15–150 µm) was used with nanostructures integrated, and even the NiCrAlY powder can be considered coarse (5–45 µm) according to Huang et al. [49]. 

The porosity in the case of TBCs is an important property, mainly because it can represent the path for corrosive particles to penetrate the ceramic coating and cause the corrosion of the underlayers, but also because the porosity was reported to permit the coating’s stress compensation during the thermal cycling [50]. 

Another unconventional method to improve the durability of TBCs was reported to be the pre-oxidation of TBCs. For example, Chen et al. [51] reported the improved durability of APS-deposited TBCs consisting of ZrO_2_–8 wt.%Y_2_O_3_/CoNiCrAlY by performing a pre-oxidation treatment that reduces the growth rate of the TGO. Moreover, Liu and Sohn [52] observed that EB-PVD ZrO_2_–7 wt.%Y_2_O_3_/NiCoCrAlY TBCs specimens with pre-oxidized bond coats had a longer lifetime than those without pre-oxidation.

**Figure 6:**
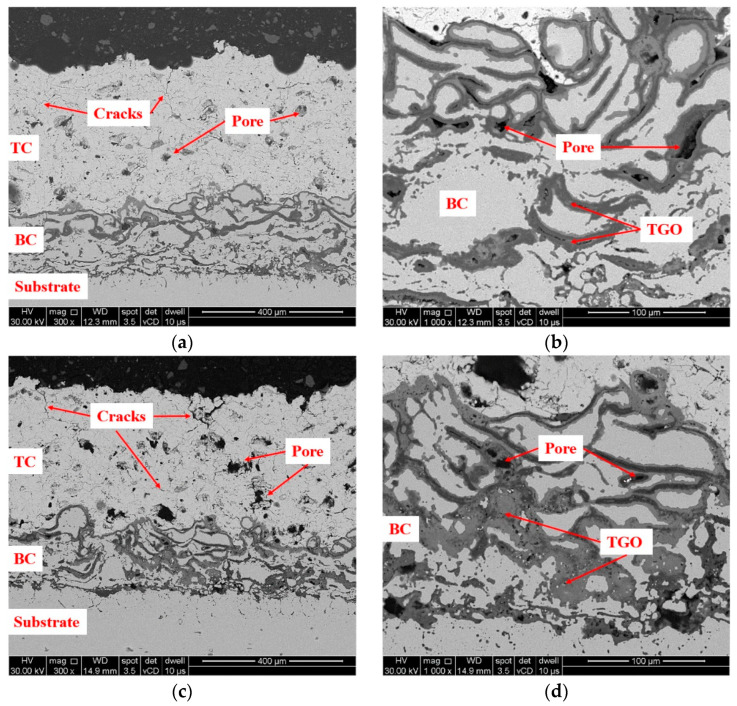
Representative SEM images in the cross section of specimens oxidized for 400 h (**a**,**b**) and 800 h (**c**,**d**).

The SEM analysis in the cross-section of specimens revealed many fine vertical cracks in the ceramic coat after 400 h of isothermal oxidation (Figure 6a,b), and even more significant cracks were observed in the ceramic coat after 800 h of isothermal oxidation, as can be observed in the SEM images from Figure 6c,d.

In addition to the SEM analysis of oxidized specimens, a quantitative analysis of porosity was performed at the bond-coat level, and the results are presented in Table 3. The prolonged oxidation ensured an increase in TGO proportions and a decrease in the porosity. The increase in TGO was caused by the infiltration of oxygen through pores and cracks in the TC, and it thereby filled all of the cavities (inter-lamellar porosities) between the bond-coat layers. In the initial state, a bond-coat porosity of 14.42% was measured, and this value decreased significantly after long high-temperature exposure as the TGO formed and filled the internal pores. 

It seems that after 800 h of exposure, the TGO layer replaced almost the entire free volume within the bond coat, consuming elements during isothermal oxidation. An increase in volume fraction of internal oxides within the bond coat was also observed by Jiang et al. [53] for APS deposited TBC with NiCoCrAlY bond coats after 799 h of 1150 °C isothermal oxidation. A difference between their results and the results obtained in the present case was observed. The previous study reported swelling of the TBC due to an increase in the thickness of the bond coat [53], while in the present case, based on the SEM images, no increase in thickness was observed. This conclusion supports the fact that no significant spallation of the TBC was produced during the present experiment. Moreover, an increase in both ceramic and bond-coat compaction was also observed after prolonged high-temperature exposure. 

The TC sinters during long hours of exposure, becoming more brittle and increasing its susceptibility to cracking, as was shown by Li et al. [54]. Sintering of the TC during high-temperature exposure is a common phenomenon, and it is one of the main reasons for TBC failure [55]. In the review of Yan et al. [55], a possible cause of this phenomenon is presented––namely, the reduction of the total interface energy of the coating. Although sintering is inevitable, Li et al. [54] mentioned that the presence of nanozones is beneficial for the overall coating performance.

As many interconnected pores were present in the bond coat where the TGO formed, it can be said that the TGO is akin to “a spider web” that bonds together several parts of the bond coat. Keeping the thickness of the TGO layer as low as possible reduces the internal stresses in the bonding layer and prevents the exfoliation of the ceramic layer. Other authors reported that the thickening of the TGO can result in spallation because it compromises the adhesion between the top layer and the bond coat [56].

The thickness of the TGO layer is kept relatively under control due to the reduced amounts of oxygen that penetrate through the ceramic layer relative to the large oxidation surface caused by porosity after deposition.

**Figure 7:**
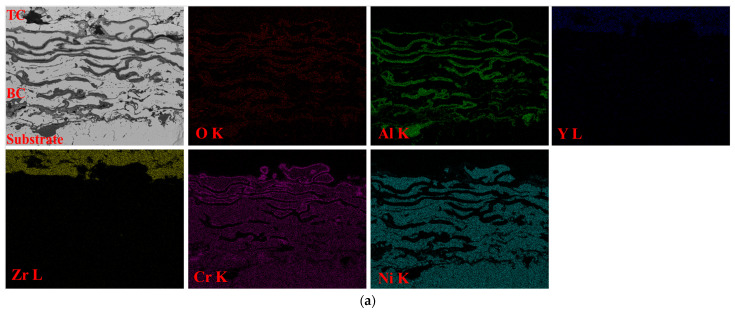

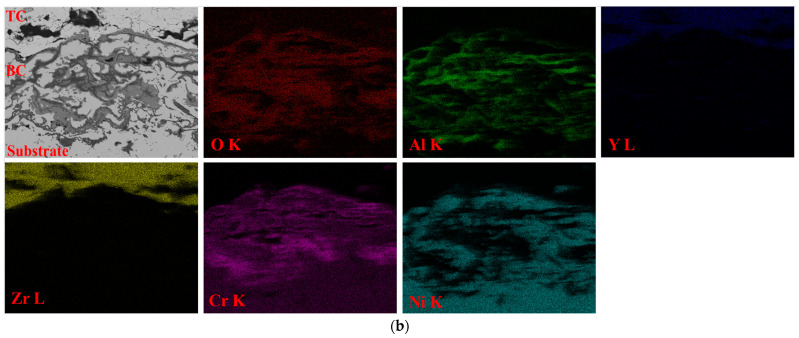
Elemental distribution maps at the bond-coat area for specimens oxidized during 400 h (**a**) and 800 h (**b**).

Regarding the chemical composition of the bond coat and the TGO layer, the elemental distribution maps obtained by EDS are presented in Figure 7 for specimens oxidized for 400 h and 800 h.

**Figure 8:**
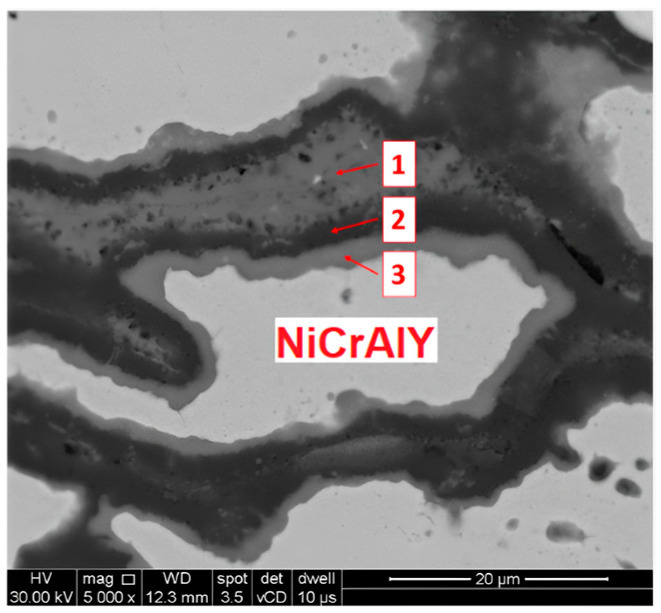
SEM image of the interface bond coat—TGO in the specimens oxidized for 800 h.

From the elemental distribution maps, it can be seen that the superior limit of the bond coat is at the interface bond-coat ceramic coat as Zr and Y are present. In addition to these elements, in the bond-coat area, high contents of Cr Al Ni and O were found, as they are constitutive elements for both the bond coat and the TGO. An increase in the oxygen content was observed after longer exposure as a higher TGO proportion developed. A punctual chemical analysis was performed by EDS in order to highlight the elemental distribution at the interface bond-coat TGO, as can be seen in Figure 8. The elements found and their ratios are both presented in Table 4.

The oxygen penetrates through pores and microcracks of the TC and reacts with Cr and Al from the bond coat to form the protective scale composed of Cr_2_O_3_ and Al_2_O_3_. Dark grey areas from Figure 8, #2 point, contain a high concentration of aluminum (present in the Al_2_O_3_ scale). However, medium gray areas from Figure 8, #3 point, consist of a high quantity of chromium and oxygen, followed by aluminum and a slight of nickel, which is a sign of mixed oxides of aluminum, chromium, and nickel. The development of (Ni,Co)(Cr,Al)_2_O_4_ spinel is possible, as was reported by other authors for TBCs [57] but also by the authors of this paper in the case of high-temperature oxidation of Ni-based superalloys [58].

At high temperatures, oxygen atoms react with metallic phases to create stable oxides due to the high affinities of oxygen with elements such as Al and Cr, and Al_2_O_3_ and Cr_2_O_3_ thereby form. The presence of high concentrations of Al and Cr in the bond coat leads to the development of oxide scales consisting of Al_2_O_3_ and Cr_2_O_3_ and also mixtures of them. The Al_2_O_3_ forms before the Cr_2_O_3_ because it has a lower Gibbs free energy value––namely, 1582.3 kJ/mol compared to 1053.1 kJ/mol [59].

Black and grey layers of TGO were observed by Ogawa [60], where the darker oxide layers consist of Al_2_O_3_ and the grey layers consist of spinels and mixed oxides. Beyond this, Ogawa observed that this mixed oxide layer influences the penetration of oxygen or has an impact on the reduction of oxygen potential. He assumed that the oxidation rate of Al_2_O_3_ is reduced concomitant to the increase in the thickness of the mixed oxides, since it is formed over the Al_2_O_3_. This assumption can be applied in the current case, too, as a high oxidation resistance of the material was registered.

Hu et al. [61] reported an oxidation mechanism for the growth of the TGO, which progresses from a bi-layered structure and reaches, in the end, a four-layered structure that includes Al_2_O_3_, Y_3_Al_5_O_12_ (YAG), NiCr_2_O_4_, and NiO.

To assess the effect of high-temperature oxidation tests on the adhesion strength of TBC layers, scratch testing was conducted on the initial TBC sample as well as after 400 h and 800 h of oxidation exposure. The results of the scratch test are presented in Table 5 along with the experimentally determined porosity of the TC based on microscopic analysis. 

As can be concluded based on the registered values, the high-temperature exposure for long periods leads to a densification of the TC. A significant difference was observed in the case of the projected cone area recorded after 400 h of exposure; a 27% reduction was recorded for this time compared with the initial value. This was also sustained by the reduction of porosity by 11% compared with the initial value. Increasing the exposure time at 1150 °C up to 800 h resulted in only a slight increase in densification compared with the value recorded at 400 h of exposure (approx. 1% reduction was registered). Even if the TC in this case is slightly denser, the longer exposure time produces larger cracks in the TC, and thus an increase in porosity was observed (a 1.74% increase in porosity compared with the initial state, and a 14.52% increase compared with the porosity recorded after 400 h of exposure). 

**Figure 9:**
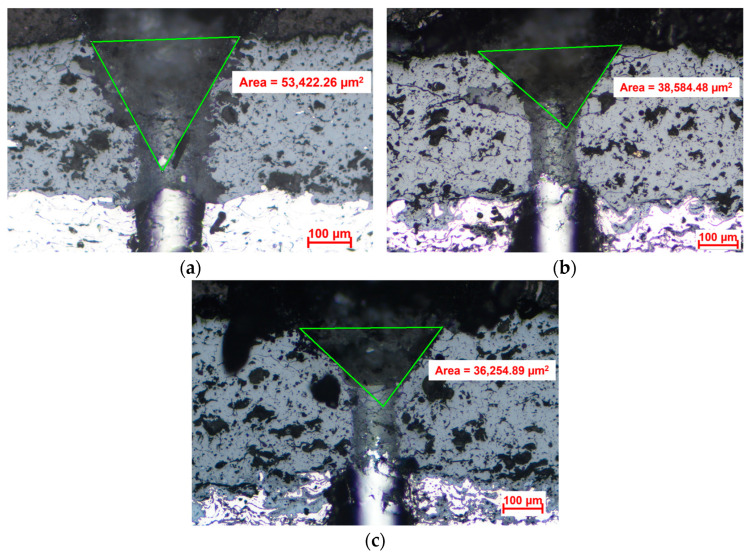
Optical microscopy images of the specimens’ cross section after scratch testing: (**a**) initial state; (**b**) after 400 h of oxidation; (**c**) after 800 h of oxidation.

Although cracks formed in the TC, after the scratch tests, no cracks were identified at the TC–BC interface, and the failure of the coating was caused by a reduction in the cohesive strength between the splats of the top coat. The development of the TGO layer throughout the volume of the bonding layer did not reduce the adhesion resistance of the TC. Figure 9 presents optical microscopy images in the specimens’ cross-section after scratch testing. 

**Figure 10:**
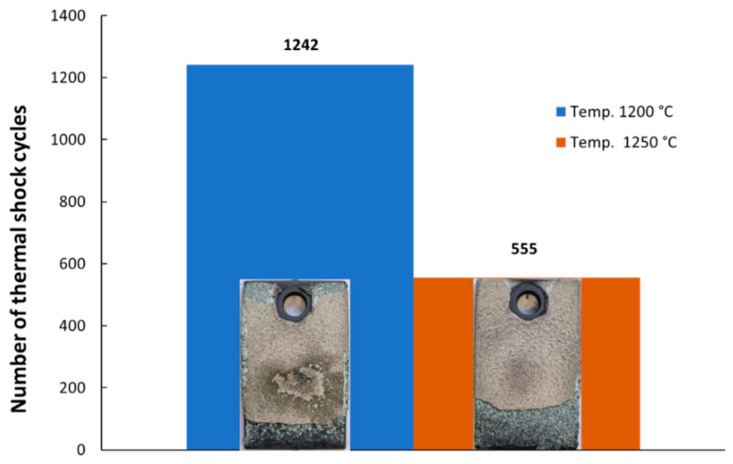
A comparative representation of the results of cyclic thermal shock tests.

Moreover, the coating resistance was verified in extreme conditions during higher temperature exposure and under thermal shock tests at 1200 °C and 1250 °C. Figure 10 presents the results of the thermal shock along with representative images of the damaged specimens. The material resisted for 1242 cycles at 1200 °C and for 555 cycles at 1250 °C. 

As shown in Figure 10, the number of thermal cycles decreased by up to 55% as the temperature increased from 1200 °C to 1250 °C. This significant decrease in thermal cycle resistance was somewhat expected due to the sintering effect of zirconia at temperatures exceeding 1200 °C. Additionally, in both cases, the exfoliation of TBC occurred primarily at the edges, and the shock testing was stopped when more than 20% exfoliation of the total area coated was observed. Studies regarding thermal shock resistance of TBCs based on YSZ were performed by other authors, too, mainly on slightly lower temperatures. For example, Zhang et al. [62] studied the thermal failure of nanostructured YSZ and NiCrAlY in comparison with conventional YSZ and NiCrAlY, and they observed that the nanostructured TBC showed a better thermal shock resistance at 1000 °C until 300 cycles were reached, and, afterwards, they increased the temperature up to 1150 °C in order to speed up the experiments. Wang et al. [63] recorded better performances for sub-micron YSZ coatings compared with nanosized YSZ in the case of thermal shock at 1100 °C. The reason behind the more rapid damage of the nanosized coating was considered to be the formation of coarse cracks during the thermal shock. Conventional YSZ coating thermal shock resistance was studied by Sun et al. [64], and they observed a good resistance through a 200-cycle thermal shock test at 1150 °C, and after 200 cycles, the bond strength decreased by 50%. Jamali et al. [65] made a comparison between the thermal shock resistance of nano-7YSZ, 15YSZ, and 5.5SYSZ (scandia, yttria doped zirconia) with a NiCrAlY used as bond coat, and they observed that the SYSZ and the 7YSZ had better performances at 1000 °C as they present a lower thermal mismatch stress between the ceramic-metallic layer. 

## 4. Conclusions

The TBCs are useful elements of the components of gas turbines, and their goal is the protection of parts at high temperatures and under severe environmental loads. The present study was focused on the performance of a nanostructured TBC deposited by APS that has a bond coat with inter-lamellar porosities that result during the manufacturing process. Based on the experimental tests, we can conclude that:

- The inter-lamellar pores produced during the deposition of the bond coat by APS do not affect the high resistance of the nanostructured TBCs in case of long-term isothermal oxidation at 1150 °C.
- The ceramic layer withstands the high temperature for 800 h of maintaining without showing major exfoliation.
- SEM analysis revealed fine cracks in the ceramic coating after 400 h of isothermal oxidation, and larger vertical cracks after 800 h of high-temperature maintaining.
- The inter-lamellar pores ensure the formation of the TGO and its spread on a larger area during high-temperature exposure, limiting the increase in thickness.
- It seems that after 800 h cumulated of maintaining at temperature, the bond coat consumes almost the entire free volume of the bond coat.
- An increase in both ceramic and bond-coat compaction was also observed after prolonged high-temperature exposure, and this was also sustained by the adhesion strength determined by the reduction of the cone-projected area during scratch testing.
- In extreme conditions, under high-temperature thermal shock cycles, the nanostructured YSZ/NiCrAlY system resisted for 1242 cycles at 1200 °C and 555 cycles at 1250 °C.

No substrate damage was recorded even if a less dense bond coat was used. It is believed that the formation of mixed oxides and the alumina layer through the inter-lamellar pores can reduce the oxygen penetration because the TGO closes the pores from the bond coat. The resistance of the coating was supported by the adhesion tests, which proved that the damage occurred at the ceramic top-coat level. Further studies will be conducted to evaluate different porosity levels of the bond coat and how they influence conventional thermal barrier coatings and other environmental coatings. Moreover, extensive studies will be realized on the corrosion resistance of thermal barrier coating with lesser density.

## Figures and Tables

**Table 1:** Chemical composition of the powders used as bond coat and top coat, determined by SEM-EDS.

Powder	Chemical Composition	O	Al	Cr	Ni	Y	Zr
Amperit 413 NiCrAlY bond coat	wt.%	8.78	10.74	24.19	59.99	4.29	-
	at. %	23.59	17.1	19.17	38.06	2.07	-
Nanox^TM^ Powder S4007 top coat	wt.%	14.7	-	-	-	8.3	77
	at. %	49.5	-	-	-	5	45.5

**Table 2:** Spraying parameters for each coating type.

Parameters	Bond Coat	Ceramic Layer	Parameters	Bond Coat	Ceramic Layer
Argon, [NLPM]	45	40	Nozzle diameter, [mm]	8	8
Hydrogen, [NLPM]	6	10.6	Injector angle, [°]	90	90
Voltage, [V]	61	70	Power Feed rate, [g/min]	50	50
Current, [A]	550	530	Spray Speed, [m/s]	1.25	1.25
Spray distance, [mm]	110	100	-	-	-

**Table 3:** Quantitative analysis performed at bond-coat level.

	Initial State	400 h	800 h
Area of thermally grown oxides [%]	0	37.11	51.84
Standard deviation	-	4.68	3.76
Porosity of the bond coat [%]	14.42	2.74	1.62
Standard deviation	1.08	0.52	0.48

**Table 4:** Chemical composition measured by EDS in the three areas from Figure 8.

Sample–Area	Chemical Composition (wt.%)			
	O	Ni	Cr	Al
#1	33.21	0.56	3.98	62.25
#2	32.98	1.26	4.22	61.54
#3	29.49	4.5	50.66	15.35

**Table 5:** The results of adhesion strength tests at 40 N.

	Initial State	400 h	800 h
Projected cone area [µm^2^]	52,497	38,201	37,786
Standard deviation	4651	4165	2475
Porosity of the top coat [%]	20.08	17.84	20.43
Standard deviation	0.47	0.45	0.52
